# Self-Guided Molecular Simulation to Enhance Concerted Motion

**DOI:** 10.3390/ijms26051969

**Published:** 2025-02-25

**Authors:** Xiongwu Wu, Bernard R. Brooks

**Affiliations:** Laboratory of Computational Biology, National Heart, Lung and Blood Institute (NHLBI), National Institutes of Health (NIH), Bethesda, MD 20892, USA

**Keywords:** molecular simulation, molecular dynamics, Langevin dynamics, algorithm, conformational search, conformational sampling

## Abstract

Self-guided (SG) molecular simulation methods, namely self-guided molecular dynamics (SGMD) and self-guided Langevin dynamics (SGLD), enhance conformational search by promoting low-frequency motion. A simple local time averaging scheme is used to extract low-frequency properties with little overhead in computing costs. For molecular processes to form ordered structures like ligand binding and protein folding, it is believed that concerted motions play crucial roles. To enhance the concerted motion in molecular systems, we propose a spatial averaging scheme to extract the concerted motion of a local region. Applying guiding forces based on spatial averaging, self-guided molecular simulations can enhance concerted motion and reach ordered structures more efficiently. Through simulations of amyloid fibril peptides, we demonstrated that the spatial averaging in self-guided Langevin dynamics results in accelerated β-sheet formation.

## 1. Introduction

Self-guided molecular dynamics (SGMD) and self-guided Langevin dynamics (SGLD), namely self-guided (SG) molecular simulation methods, are developed to enhance conformational search and sampling. Many applications can be found in the review article [1]. Most enhanced conformational sampling methods rely on certain biased potentials to help overcome energy barriers, such as metadynamics [2], accelerated molecular dynamics [3], and self-adapted accelerated molecular dynamics [4]. SG methods do not rely on a priori energy barrier information to enhance sampling. Instead, they achieve an enhanced conformational search through promoting low-frequency motion. Low-frequency motion is a well-known limiting factor for conformational searching and sampling. Low-frequency momentum is related to diffusional motion, which is essential for diffusion-controlled conformational change, while low-frequency force is related to a smoothed energy surface, which is responsible for energy-barrier-controlled conformational change.

SG methods are unique in their ability to extract low-frequency properties through a simple local averaging scheme, which does not incur a significant overhead in computing costs. This local averaging scheme produces running time average properties. Guiding forces based on these local time averages would promote low-frequency motion. There are many types of motion in molecular systems, such as thermal motion, diffusion, binding, folding, etc. Most motion is random in nature, whereas some is concerted and leads to more ordered structures. Concerted motions are believed to be essential for structure changes in molecular systems, such as crystallization, ligand binding, protein folding, etc. To identify the concerted motion, we propose a spatial averaging scheme in addition to the local time averaging. Guiding forces based on the concerted motion can be used to enhance such motion in SG simulations.

The concept and implementation of the spatial averaging scheme are described in the method section. A demonstration simulation of β-sheet formation is presented as an application of the method. The spatial averaging presented here has been implemented in current versions of CHARMM [5] and Amber [6], which are molecular simulation and modeling packages widely used in computational studies.

## 2. Results and Discussion

To demonstrate the effect of spatial averaging in SGLD simulations, we chose a system of amyloid fibrils to simulate the formation of β-sheets. Aggregation of amyloid fibrils has been identified as a leading cause of Alzheimer’s disease. Extensive experimental and computational studies have been carried out to study the mechanism of amyloid fibril aggregation. Computer simulation is a valuable approach for studying this mechanism at the atomic level. However, due to the large conformational space and rugged energy landscape, molecular simulation of amyloid fibril is often trapped into local minimum conformations. Therefore, an enhanced conformational search technique is needed to efficiently simulate the aggregation process.

**Figure 1:**
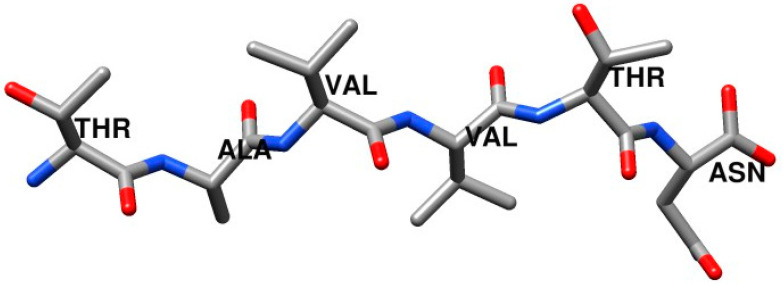
The amyloid-driving segments of the tetrameric thyroxine transport protein transthyretin (TTR), ^119^TAVVTN^124^. Only heavy atoms are shown. C, O, and N atoms are colored as grey, red, and blue, respectively.

To illustrate the effects of spatial averaging, we performed LD, SGLD, and SGLD with spatial averaging. The amyloid-driving segments of the tetrameric thyroxine transport protein transthyretin (TTR) [7] were chosen for their simplicity. Figure 1 shows the structure of the amyloid-driving segments of TTR, ^119^TAVVTN^124^.

Instead of using a periodic boundary condition, we construct an EMAP box to limit the simulation molecules from flying away. An EMAP box is a map object that has densities at the boundary and which interacts with simulation objects with a map potential [8]:

(1) $$E_{\mathrm{map}}=-c_{\mathrm{map}}\sum_{a}^{N}m_{a}\hat{\rho }(x_{a},y_{a},z_{a})$$

$\hat{\rho }(x_{a},y_{a},z_{a})$ is the density at position $\left(x_{a},y_{a},z_{a}\right)$. The restraint constant, $C_{\mathrm{map}}$, sets the strength of the map-restraint. The units of atomic mass, $m_{a}$ and $c_{\mathrm{map}}$ are g/mol and kcal/g, respectively. The EMAP boundary is generated in the simulation, and no EMAP file is needed. However, existing boundary EMAP files can be used if users want. In this work, the boundary map is a 20 × 20 × 20 Å^3^ cube with a grid space of 4 Å. A negative restraint constant, $c_{\mathrm{map}}=-1.0\;g/\mathrm{mol}$, is used to repel atoms from approaching the boundary.

The simulations are performed with a friction constant of γ=1/ps. For SGLD simulations, the guiding factor is set to λ=0.2, and the local average time is $t_{L}=0.2/\mathrm{ps}$. For the spatial averaging, we chose $d_{L}=5Å$. The AMBER force field ff19sb [9] is used for energy calculation. The generalized Born solvation model is used to calculate the solvation energy [10,11]. AMBER simulation program PMEMD [6] is used to perform the simulation. The SGLD method and its spatial averaging scheme, as well as the EMAP functions, have been implemented in current versions of CHARMM [5] and Amber [6] and are available in either Sander or PMEMD.

**Figure 2:**
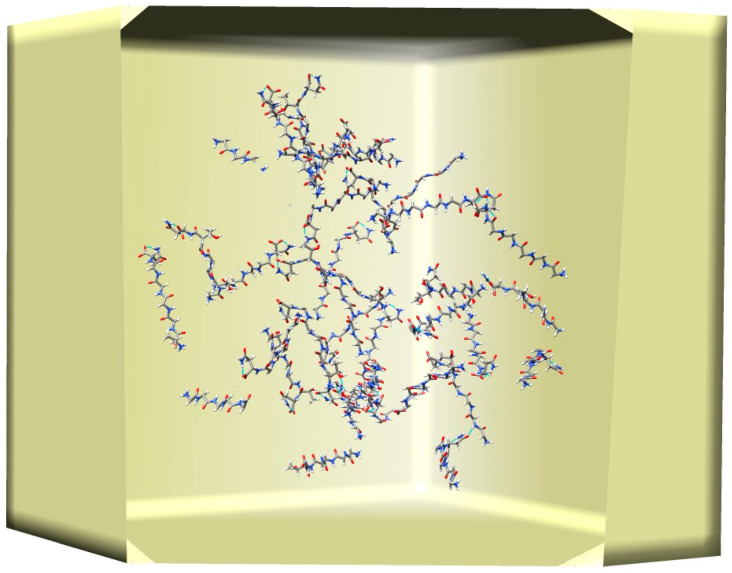
The initial conformation of the simulations, where 40 TAVVTN peptides are randomly placed in a 20 × 20 × 20 Å^3^ cubic EMAP boundary box. Only heavy atoms are shown. C, O, and N atoms are colored as grey, red, and blue, respectively.

Figure 2 shows the initial conformation for the simulations, where 40 TAVVTN peptides are randomly placed in the EMAP boundary box. Starting from this initial conformation, three simulations, one with Langevin dynamics (LD), one with SGLD, and one with SGLD and spatial averaging, are carried out.

**Figure 3:**
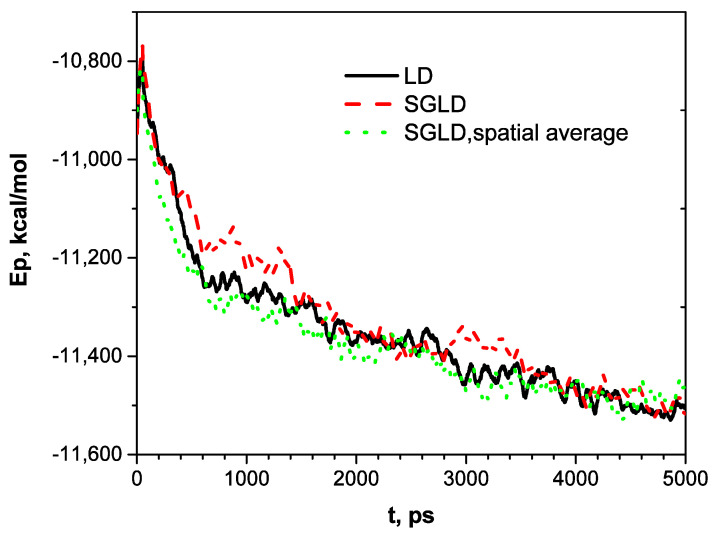
Potential energies along the LD, SGLD, and SGLD/spatial averaging simulation trajectories.

Figure 3 shows the potential energy profiles along the three trajectories. As can be seen, potential energies decrease along rugged curves. The SGLD curve shows frequent energy jumps, indicating its guiding forces can lead to conformational changes with significant energy increases. With spatial averaging, SGLD has a faster energy decrease at the beginning than LD. In 5000 ps, all three simulations reach similar energy levels.

**Figure 4:**
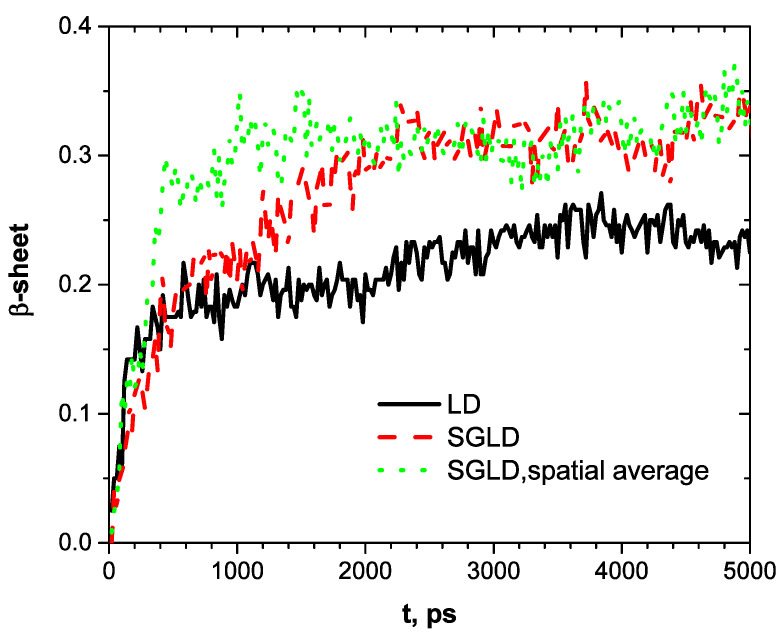
β-sheet fractions along the LD, SGLD, and SGLD/spatial averaging simulation trajectories.

Figure 4 shows the conformational changes, as measured by β-strand fractions. The directory of secondary structure of proteins (DSSP) [12] is employed for the analysis. Starting from a fraction of 0, the LD simulation reaches a fraction of 0.18 in the first 400 ps, then slowly grows to 0.22 and fluctuates around this level. The SGLD simulation reaches 0.2 after 600 ps, a little bit slower than the LD simulation, but grows to 0.3 after 2200 ps and fluctuates around this value after that. With spatial averaging, SGLD quickly reaches 0.3 in 600 ps and fluctuates around this level afterward. These simulation results show that the LD simulation quickly descends to a local minimum conformation with a β-strand fraction of 0.18 and is trapped there for a long time with slow conformational changes. The SGLD simulation can quickly overcome energy barriers and reach states with high β-strand fractions. With spatial averaging, the SGLD quickly reaches states with high β-strand fractions, faster than the SGLD simulation without spatial averaging. In other words, the spatial averaging promoted the formation of the β-sheet conformation. From the fluctuations in Figure 4, one can see that SGLD and SGLD/spatial average can result in large structure changes that would help a system overcome energy barriers. SG simulations will not focus on any specific structure but will move faster from one structure to another structure.

**Figure 5:**
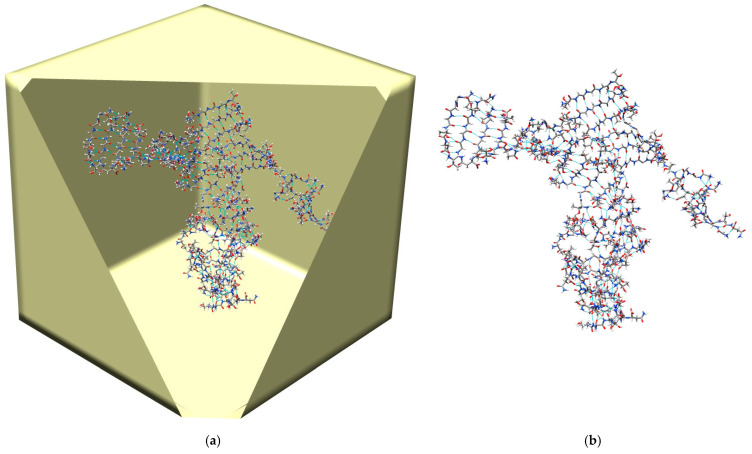
A typical β-sheet conformation formed in the SGLD/spatial averaging simulation. (**a**) The β-sheet inside the EMAP box; (**b**) the β-sheet alone. Only heavy atoms are shown. C, O, and N atoms are colored as grey, red, and blue, respectively.

Figure 5 illustrates a typical β-sheet conformation reached in the SGLD/spatial averaging simulation. Clearly, we can see several β-sheets formed by β-strands of the peptides. These β-sheets formation processes provide us opportunities to analyze the atomic details of amyloid fibril aggregation, which is beyond the scope of this paper and will be discussed in future studies.

The acceleration in β-sheet formation demonstrates enhanced concerted motion due to spatial averaging. Concerted motion is different in structured regions and unstructured regions. Motion in intrinsically disordered proteins will be enhanced by regular time local averaging but may not be enhanced by spatial averaging because, in unstructured regions, concerted motion may be weak.

The EMAP boundary box used in these simulations has significant effects on the simulation results. First, the size of the boundary limits the conformational space of the simulation system. Second, the strength of the boundary potential may control how fast a molecule bounces back when hitting the boundary. Third, the shape of the boundary may limit the geometry of the aggregation. Therefore, the boundary box should be large enough to minimize these effects and small enough to reduce the conformational search space.

The above demonstration simulations show the differences among LD, SGLD, and SGLD/spatial averaging. Thorough analysis and comparison will be conducted in our future publications.

## 3. Materials and Methods

### 3.1. The Equation of Self-Guided Motion

The equation of generalized self-guided motion [13] has the following form:

(2) $$\dot{p}=F+g$$

Here, **p** is the atom’s momentum, and $\dot{p}$ is its time derivative. F is the apparent force acting on the atom, including all contributions such as molecular interactions, constraint forces from SHAKE [14] if present, friction forces and random forces in Langevin dynamics, velocity scaling for constant temperature simulations, etc. g is the guiding force that is based on the local average momentum, $\tilde{p}$, and the local average forces, $\tilde{F}$ and $\tilde{\tilde{F}}$:

(3) $$g=\lambda \xi \tilde{p}+\mu (\tilde{F}-\tilde{\tilde{F}})$$

λ, and force guiding factor, μ. The low-frequency characteristics of the guiding forces provide a way to alter the low-frequency motion of the simulation system. The parameter ξ represents an apparent friction constant of the simulation system, which is estimated during the SG simulation as follows:

(4) $$\xi =-\frac{<\left(\tilde{F}-\tilde{\tilde{F}}\right)\cdot \tilde{p}>}{<\tilde{p}\cdot \tilde{p}>}$$

The tilde “~” on a property represents its local time average, which is calculated during a simulation using the following scheme along trajectories:

(5) $$\tilde{P}\left(t\right)=\frac{1}{t_{L}}\int_{-\infty }^{t}{Pe}^{-\frac{t-\tau }{t_{L}}}d\tau \approx \left(1-\frac{\delta t}{t_{L}}\right)\tilde{P}\left(t-\delta t\right)+\frac{\delta t}{t_{L}}P(t)$$

Here, P is any time-dependent property, and $\tilde{P}$ is its local time average. Likewise, $\tilde{\tilde{P}}$ is the local time average of $\tilde{P}$. $t_{L}$ is the local average time, which defines a frequency threshold, $1/t_{L}$. Properties with frequencies higher than this threshold are filtered down, while those with lower frequencies are mostly kept by the averaging.

While both guiding forces can influence the low-frequency motion, the momentum guiding force (when λ>0) would promote diffusion-controlled conformational search, and the force guiding force (when μ<0) would promote energy-barrier-controlled conformational search. The two guiding effects would cancel out each other when the guiding factors satisfy the following relation:

(6) $$\lambda_{\mu }={\left(1+\mu \right)}^{2}-\frac{1}{1+\mu }$$

We call $\lambda_{\mu }$ the balanced momentum guiding factor of μ. 

### 3.2. The Spatial Averaging Scheme

The idea of spatial averaging is to extract common motions shared in a local region. Vibration, shaking, random walk, etc., are not shared motions and will be canceled out in spatial averaging. Complexes, folded structures, crystalline, etc., will have shared motion by their atoms. The understanding that these shared motions are essential for the formation of these structures leads us to develop the spatial averaging scheme.

We define the spatial averaging by the following formula:

(7) $${\hat{p}}_{i}=m_{i}\frac{\sum_{{j\in \Omega }_{i}}{\tilde{p}}_{j}w_{ij}}{\sum_{{j\in \Omega }_{i}}m_{j}w_{ij}}$$

Here, we use ${\hat{p}}_{i}$ to represent the spatial averaging of property $p_{i}$. $w_{ij}$ is a weighting factor for spatial averaging. Typically, $w_{ij}=1$. $\Omega_{i}$ defines the spatial range of atom *i*, which can be chemical structures or a region in space. Several spatial range types are available in the CHARMM [5] and Amber [6] implementations:

Type 1: No spatial averaging. $\Omega_{i}=i$

Type 2: Averaging over bond-length and bond-angle atoms. $\Omega_{i}$ contains atoms strongly bonded with atom *i*.

Type 3: Averaging over bond-length, bond-angle, and dihedral angle atoms. $\Omega_{i}$ contains atoms in its local chemical structure.

Type 4: Averaging over all atoms within a local distance, $d_{L}$. $\Omega_{i}$ contains all atoms within a certain distance.

**Figure 6:**
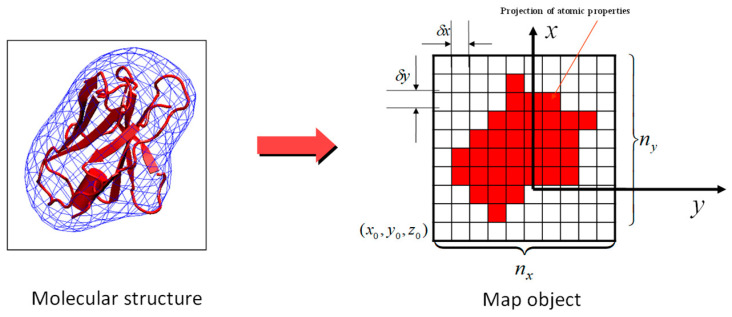
Map representation of local spatial averages. The intervals between grid points are $\delta x=\delta y=\delta z=d_{L}$. Atom-occupied grids are colored red. Local average momentum and forces of atoms are projected to the grid points directly surrounding them.

For type 4 spatial averaging, we adopted EMAP functions [8,15] implemented in AMBER [6] and CHARMM [5] for calculation. As shown in Figure 6, atomic properties (local average momentums and forces) are distributed over grid points to form a map of objects. The interval between grid points is $d_{L}$. $\Omega_{i}$ contains all grid points directly surrounding atom *i*.

Introducing the local spatial averaging only changes how the local average properties are calculated but will not change the equation of the self-guided motion. Therefore, we expect that the simulation can be reweighted using the SG method described previously [13].

### 3.3. A Leap-Frog Algorithm for SGMD/SGLD with Spatial Averaging

Here, we present a leap-frog algorithm to implement the SGMD/SGLD simulation method with the spatial averaging scheme. This algorithm needs vector arrays to store $\tilde{r}$, $\tilde{\tilde{r}}$, and $\tilde{p}$ and uses these arrays to calculate all other quantities. In addition, an EMAP object is needed for type 4 spatial averaging. The following algorithm has been implemented in current versions of CHARMM [5] (49b1) and Amber [6] (2024). Currently, SGLD implementations support the PMEMD GPU version, but SGLD/spatial average is only implemented in the CPU version. We will implement it in the GPU version only after being widely tested.

(1) At each time step, t, calculate the potential energy, $E_{p}$, and forces, f, which include the random forces in Langevin dynamics.

(2) Calculate the local time average properties.

Local average energies:

(8) $${\tilde{E}}_{p}(t)=\left(1-\frac{\delta t}{t_{L}}\right){\tilde{E}}_{p}\left(t-\delta t\right)+\frac{\delta t}{t_{L}}E_{p}\left(t\right)$$

(9) $${\tilde{\tilde{E}}}_{p}(t)=\left(1-\frac{\delta t}{t_{L}}\right){\tilde{\tilde{E}}}_{p}\left(t-\delta t\right)+\frac{\delta t}{t_{L}}{\tilde{E}}_{p}\left(t\right)$$

Local average positions:

(10) $$\tilde{r}\left(t\right)=\left(1-\frac{\delta t}{t_{L}}\right)\tilde{r}\left(t-\delta t\right)+\frac{\delta t}{t_{L}}r\left(t\right)$$

(11) $$\tilde{\tilde{r}}\left(t\right)=\left(1-\frac{\delta t}{t_{L}}\right)\tilde{\tilde{r}}\left(t-\delta t\right)+\frac{\delta t}{t_{L}}\tilde{r}\left(t\right)$$

Other local average properties, $\tilde{p}$ and $\tilde{F}$ and $\tilde{\tilde{F}}$ are calculated from $\tilde{r}\left(t\right)$ and $\tilde{\tilde{r}}\left(t\right)$:

(12) $$\tilde{p}\left(t\right)=\frac{m}{t_{L}}(r\left(t\right)-\tilde{r}\left(t\right))$$

(13) $$p\left(t\right)=\tilde{p}\left(t-\delta t\right)+\frac{t_{L}}{\delta t}(\tilde{p}\left(t\right)-\tilde{p}\left(t-\delta t\right))$$

(14) $$\tilde{F}\left(t\right)=\frac{1}{t_{L}}p\left(t\right)-\frac{m}{t_{L}^{2}}(r\left(t\right)-\tilde{r}\left(t\right))$$

(15) $$\tilde{\tilde{F}}\left(t\right)=\frac{m}{t_{L}^{2}}(r\left(t\right)-2\tilde{r}\left(t\right)+\tilde{\tilde{r}}\left(t\right))$$

Therefore, only the local average positions, $\tilde{r}\left(t\right)$ and $\tilde{\tilde{r}}\left(t\right)$, and $\tilde{p}\left(t\right)$, need to be stored during an SG simulation.

(3) Calculate local spatial averaging properties.

If the spatial averaging type is 1, the spatial averages will be the same as local time averages.

If the spatial averaging type is 2 or 3, the properties of bonded atoms are summed up for averaging according to Equation (7).

If the spatial averaging type is 4, project m, $\tilde{p}\left(t\right)$, and $\tilde{∆F}=\tilde{F}\left(t\right)-\tilde{\tilde{F}}\left(t\right)$ over the grid points around each atom. The grid interval between grid points is set to the spatial averaging distance, $d_{L}$.

(16) $${\hat{v}}_{g}=\frac{\sum_{{j\in \Omega }_{i}}\left(1-\left|x_{jg}\right|)(1-\left|y_{jg}\right|)(1-\left|z_{jg}\right|\right){\tilde{p}}_{j}}{\sum_{{j\in \Omega }_{i}}\left(1-\left|x_{jg}\right|)(1-\left|y_{jg}\right|)(1-\left|z_{jg}\right|\right)m_{j}}$$

(17) $${\hat{a}}_{g}=\frac{\sum_{{j\in \Omega }_{i}}\left(1-\left|x_{jg}\right|)(1-\left|y_{jg}\right|)(1-\left|z_{jg}\right|\right){\tilde{∆F}}_{j}}{\sum_{{j\in \Omega }_{i}}\left(1-\left|x_{jg}\right|)(1-\left|y_{jg}\right|)(1-\left|z_{jg}\right|\right)m_{j}}$$

Here, $x_{jg}=\frac{x_{g}-x_{j}}{d_{L}}$, $y_{jg}=\frac{y_{g}-y}{d_{L}}$, $z_{jg}=\frac{z_{g}-z_{j}}{d_{L}}$; g represents the index of the grid points directly surrounding the atom.

The spatial averagings for each atom are calculated by interpolating from the grid points around the atom.

(18) $${\hat{p}}_{i}=m_{i}\sum_{{g\in \Omega }_{i}}\left(1-\left|x_{ig}\right|)(1-\left|y_{ig}\right|)(1-\left|z_{ig}\right|\right){\hat{v}}_{g}$$

(19) $${\hat{∆F}}_{i}=m_{i}\sum_{{g\in \Omega }_{i}}\left(1-\left|x_{ig}\right|)(1-\left|y_{ig}\right|)(1-\left|z_{ig}\right|\right){\hat{a}}_{g}$$

(4) Calculate apparent friction constants, ξ.

We need ensemble averages of $\hat{∆F}\cdot \hat{p}$ and $\hat{p}\cdot \hat{p}$ to calculate the apparent friction constant of each atom according to Equation (4). To be able to calculate them on the fly, we replace the ensemble averages by long-time local averages. An average time, $t_{avg}$, typically 10 times the local average time, $t_{L}$, is defined for this purpose. Two scalar arrays, FP and PP, are used to store the averages.

(20) $$FP(t)=\left(1-\frac{\delta t}{t_{avg}}\right)FP(t-\delta t)+\frac{\delta t}{t_{avg}}\hat{∆F}\cdot \hat{p}$$

(21) $$PP(t)=\left(1-\frac{\delta t}{t_{avg}}\right)PP(t-\delta t)+\frac{\delta t}{t_{avg}}\hat{p}\cdot \hat{p}$$

(22) $$\xi =-\frac{FP(t)}{PP(t)}$$

The averages and friction constants are calculated for each atom. Alternatively, the apparent friction constants of atoms can be calculated from previous simulations and read in at the beginning of a simulation to avoid their calculation on the fly.

(5) Calculate the guiding forces:

(23) $$g=\mu \hat{∆F}+\lambda \xi \hat{p}$$

(6) Calculate the energy conservation scaling factor:

An energy conservation scaling factor, η, is used to cancel energy input due to the guiding force. Previously, a uniform energy conservation factor was used for all atoms. Here, we use atom-specific scaling factors, and their calculations need no information from other atoms, therefore saving inter-processor communication in parallel computing.

(24) $$\eta =\frac{(2+\gamma \delta t)gp_{0}}{{2p}_{0}^{2}-gp_{0}\delta t}$$

Here,

(25) $$p_{0}=mv\left(t-\frac{\delta t}{2}\right)+\left(f+g-\gamma mv\left(t-\frac{\delta t}{2}\right)\right)\frac{\delta t}{2}$$

(7) Calculate velocities at $t+\frac{\delta t}{2}$:

(26) $$v\left(t+\frac{\delta t}{2}\right)=\frac{\left(1-\frac{(\gamma +\eta )\delta t}{2}\right)}{1+\frac{(\gamma +\eta )\delta t}{2}}v\left(t-\frac{\delta t}{2}\right)+\frac{f+g}{1+\frac{(\gamma +\eta )\delta t}{2}}\frac{\delta t}{m}$$

Please note, for SGLD, γ is the friction constant, and **f** is the interaction force plus the random force, and for SGMD, γ = 0, and there is no random force in **f**.

(8) Calculate positions at t+δt:

(27) $$r\left(t+\delta t\right)=r\left(t\right)+v\left(t+\frac{\delta t}{2}\right)\delta t$$

(9) Go back to step (1) and repeat the above steps for the next time step.

## 4. Concluding Remarks

The self-guided molecular simulation method was motivated to overcome randomness in conformational search and to accelerate the process to reach global minimum states. A local averaging scheme is the main characteristic for extracting self-guiding information during a simulation. Previously, the local time averaging scheme has focused on enhancing conformational search and sampling by promoting low-frequency motion. This work explores the spatial averaging scheme with the goal of enhancing concerted motion in simulation systems to facilitate the formation of ordered structures.

This work demonstrates that spatial averaging promotes the β-sheet formation of amyloid fibril peptides, as compared with regular Langevin dynamics and local time-average-based self-guided Langevin dynamics. An extensive examination of the behavior of spatial averaging will be conducted with more systems to fully understand its effects and optimal parameters.

With this new development, we expect SGMD/SGLD will have more options to study problems such as ligand docking and protein folding, where studying ordered structures is the goal.

## Data Availability

Data are contained within the article.
